# 1785. Infectious Etiologies for Post-Donation Deferrals in a Military Blood Donation Center

**DOI:** 10.1093/ofid/ofad500.1614

**Published:** 2023-11-27

**Authors:** Somin Kwon, Brian Casleton, Glorimar Rivera, Melita Gella, Erin Winkler, John Kieffer, Angela Osuna, Theresa Casey, Heather Yun, Joseph Marcus

**Affiliations:** Uniformed Services University of the Health Sciences, Potomac, Maryland; Armed Services Blood Bank San Antonio, San Antonio, Texas; Armed Services Blood Bank San Antonio, San Antonio, Texas; Armed Services Blood Bank San Antonio, San Antonio, Texas; BAMC, San Antonio, Texas; BAMC, San Antonio, Texas; BAMC, San Antonio, Texas; BAMC, San Antonio, Texas; Brooke Army Medical Center, TX; Brooke Army Medical Center, TX

## Abstract

**Background:**

The burden of transfusion-transmitted infections (TTIs) among blood recipients remains low due to strict blood donor selection as well as extensive post-donation screening. However, the military has the unique challenge of providing blood in austere environments with limited testing capabilities. This study evaluates infectious etiologies of deferred blood donors at a large military blood donation center.

**Methods:**

All blood donors at the Armed Service Blood Bank Center-San Antonio between 2017-2022 with positive post-donation screening tests for hepatitis C (HCV), hepatitis B (HBV), HIV, HTLV-1/2, Zika, West Nile virus (WNV), *T. cruzi, T. pallidum,* or Babesia were evaluated. Patients were deferred from donating based on FDA criteria including a positive screen with positive confirmatory testing, a positive screen with indeterminate confirmatory testing, and a second occurrence of a positive screen with negative confirmatory testing.

**Results:**

Of the 89,459 blood donors during the study period, 213 (238 per 100,000 donors) met FDA criteria for deferral. *T. pallidum* (n=45, 50.3 per 100,000), HCV (n=34, 38.0 per 100,000), and HBV (n=19, 21.2 per 100,000) were the most common infections seen in those with both positive screening and positive confirmatory testing. The majority of HIV (95%), Chagas (78%), HTLV-1/2 (50%) deferrals were due to indeterminate confirmatory tests following initial positive screens. The majority of deferrals for HBV were for a second occurrence of a positive screening test despite negative confirmatory testing.

**Conclusion:**

The rates of deferral due to positive post-donation screening for infectious diseases were low in this military cohort. In environments with limited resources, donor testing in service members should focus on HBV, HCV, and *T. pallidum* as the most common pathogens. Our findings also highlight the need for better diagnostics in screening donors for HIV, Chagas, and HTLV-1/2 due to frequent indeterminate confirmatory tests.

**Disclosures:**

**All Authors**: No reported disclosures

